# Pure tone and speech audiometry of presbyacusis in a tertiary hospital in Kaduna, Nigeria: a cross-sectional study

**DOI:** 10.11604/pamj.2024.48.184.35404

**Published:** 2024-08-22

**Authors:** Usman Ibrahim Dansani, Abubakar Danjuma Salisu, Musa Thomas Samdi, Garba Mainasara Mohammed

**Affiliations:** 1 Department of Clinical Services, National Ear Care Centre Kaduna, Kaduna, Nigeria,; 2 Department of Ear, Nose, and Throat (ENT), Aminu Kano Teaching Hospital, Bayero University Kano, Kano, Nigeria,; 3 Department of Ear, Nose, and Throat (ENT), Barau Dikko Teaching Hospital, Kaduna State University, Kaduna, Nigeria

**Keywords:** Presbyacusis, age-related, hearing, loss, pure tone, speech, audiometry

## Abstract

**Introduction:**

presbyacusis is age-related hearing loss, manifesting as a bilateral symmetrical sensorineural hearing loss of adult-onset. It has different audiometric configurations and is associated with a decline in speech discrimination sensitivity. The objectives of the study were to determine the degree of hearing loss; types of presbyacusis; and speech discrimination score (SDS) of presbyacusics and to compare the SDS in different types of presbyacusis in patients seen at a tertiary hospital in Kaduna, Nigeria.

**Methods:**

a descriptive cross-sectional study, involving 41 presbyacusics seen at the ear, nose and throat (ENT) clinic of a tertiary hospital in Kaduna. Participants had clinical evaluation; pure tone and speech audiometry with clinical audiometer Avant A2D+ Med Rx, USA. Results were analysed using SPSS version 20. Analysis of variance (ANOVA) and t-test were used to evaluate the difference between Speech discrimination scores in different types of presbyacusis, the level of significance was set at a p-value < 0.05.

**Results:**

hearing threshold ranged from 26.3-107.5 dBHL, mean of 55.7±18.2 dBHL. Moderate hearing loss (46.3%) was the most prevalent degree of hearing loss. Neural presbyacusis (28.8%) was the commonest. Speech reception threshold (SRT) ranged from 30-85dBHL mean of 61.8±14.9 dBHL, and SDS ranged from 0-98% mean of 69.38±20.0%. SDS of sensory presbyacusis was statistically higher than cochlear-conductive, and neural presbyacusis (p-value 0.026 and 0.001 respectively) while the metabolic was higher than neural presbyacusis (p-value 0.005).

**Conclusion:**

neural presbyacusis was the commonest, moderate hearing loss was the most prevalent degree of hearing loss and sensory presbyacusis had the best SDS.

## Introduction

Age-related hearing loss is a common sensory degenerative disorder with deleterious socioeconomic and psychological consequences [1]. Presbyacusis is a diagnosis of exclusion where other causes of hearing loss have been excluded. It is a generic disease with a complex aetiology involving both genetic predisposition and environmental factors. Schucknecht [2] described four types: sensory, neural, metabolic, and cochlear conductive presbyacusis. Researchers have recently described two more, i.e. mixed and indeterminate types [3].

Neural presbyacusis results due to a decrease in the population of auditory neurons in the cochlear and auditory nerve, characterized by moderate sloping high-frequency hearing loss with poor speech discrimination; sensory presbyacusis occurs due to atrophy of the organ of Corti, with the characteristic audiogram of a steep slope, high-frequency hearing loss; metabolic (strial) presbyacusis results due to degeneration of strial vascular, and has a flat audiogram; mechanical (cochlear conductive) presbyacusis results due to stiffening of the basilar membrane, it has a gentle sloping high-frequency sensorineural hearing loss [2,3].

Lasisi *et al*. [4] in Ibadan found a prevalence of hearing impairment among the elderly to be 6.1% (79) among 1302 respondents and 2.5% (32) of the 1302 respondents with hearing loss had presbyacusis. Onotai and Ebong [5] reported presbyacusis made up 10.48% of the cases of sensorineural hearing loss. Amedofu *et al*. [6] found presbyacusis to be 10.5% of cases of hearing loss. Al-Ruwali and Hagr [7] reported a prevalence of presbyacusis of 17.35%. Pure tone audiometry (PTA) is a psychoacoustic test that identifies an individual hearing threshold, it helps in the diagnosis of presbyacusis by demonstrating a bilateral symmetrical sensorineural hearing loss it also helps in distinguishing the Schuknecht pathological types of presbyacusis based on their characteristic audiogram [3].

Speech audiometry includes an assessment of speech discrimination score (SDS) which measures the percentage of phonetically balanced words correctly heard by the patient at 30-40 dB above the speech reception threshold (SRT) which is the minimum intensity at which 50% of spondee are repeated correctly by a person. In neural presbyacusis, there is a poor speech discrimination score while the other types of presbyacusis have a good speech discrimination score [3]. The objectives of the study were to determine the degree of hearing loss; types of presbyacusis; and speech discrimination score (SDS) of presbyacusics and to compare the SDS in different types of presbyacusis in patients seen at a tertiary hospital in Kaduna, Nigeria. The research questions were: what are the pure tone audiometric findings, and what are the speech audiometric findings of participants with presbyacusis seen at the ENT clinic of a tertiary hospital in Kaduna, Nigeria?

## Methods

**Study design:** a descriptive cross-sectional study of patients with presbyacusis seen at the ENT clinic of a tertiary ENT Hospital in Kaduna, Nigeria.

**Setting:** the study was done in the National Ear Care Centre (NECC), located in Kaduna metropolis, the capital of Kaduna state, North-west Nigeria. Kaduna city is cosmopolitan located at longitude 7° 31'23"E, latitude 10° 26'25"N, with an estimated population of 1.3 million people from diverse ethnic groups. National Ear Care Centre is a 50-bed tertiary hospital, that attends to an average of 12,300 new patients per year with various ear nose and throat diseases. The clinical services section has an ENT clinic, general out-patient department, accident and emergency, audiology, medical laboratory, radiology, pharmacy and medical records departments. It is an accredited centre for ENT residency training by the West African College of Surgeons and the National Postgraduate Medical College of Nigeria. The centre also has a School of Post-Basic ORL Nursing, a School of Audiology and School of Health Information Management. The test population included all patients with diagnosis of presbyacusis being managed in the ENT Clinic of NECC, Kaduna seen between May and November 2019 who consented to be part of the study.

**Participants:** patients with presbyacusis seen at the ENT clinic of the National Ear Care Centre aged 45 years and above were eligible for selection during the period of the study.

**Variables:** the variables were demographic and audiometric. The demographic variables included age, sex and ethnic group while the audiometric variables were pure tone audiometry, and speech audiometry (speech reception threshold and speech discrimination score). The outcome of the study was the hearing threshold measured in decibel hearing level (dBHL), configuration of the audiogram which signifies the type of presbyacusis, and degree of hearing loss; the speech reception threshold measured in dBHL and speech discrimination score measured in percentage.

### Data sources and measurement

**Data collection tools:** pure tone audiometry and speech audiometry were done using the clinical audiometer model Avant A2D+ MedRx, USA. The procedures were done at the audiology department of National Ear Care Centre in a soundproof room by the researchers and audiology technicians. Demographic and clinical findings were entered into a structured questionnaire, and audiometric findings were recorded into an audiogram.

**Data collection:** all consecutive participants who met the inclusion criteria were finally recruited until the study population was obtained. The participants had pure tone and Speech audiometry. Pure tone audiometry (air and bone conduction) was done using the modified Hughson-Westlake technique [8]. The result was plotted on an audiogram and the pure tone average was calculated using the hearing level at frequency values 500, 1000, 2000 and 4000 hertz for the air conduction. Hearing impairment was classified according to WHO classification [9] the clinical audiometer was switched to speech audiometry mode, and speech reception threshold and speech discrimination score were tested.

The speech reception threshold (SRT) was assessed first by delivering to each ear through a headphone a set of spondee (two-syllable words with equal stress) the English spondee word list form A and B was used which was built in the Avant A2D+ Med Rx clinical audiometer; starting from the hearing threshold and increasing the intensity by 5 dB steps until 50% of the words were correctly heard, that was the SRT, (for participants that correctly heard 50% at hearing threshold it was reduced by 5 dB until the SRT was ascertained) [5]. The speech discrimination score was measured by increasing the intensity of sound by 30 dB above the SRT of the subject, at that intensity 50 phonetically balanced (PB) English monosyllabic words (W-22 list one form A) were delivered to each ear via headphone [5]. The speech discrimination score was categorized as very poor (≤52%), poor (54-64%), fair (66-76%), good (78-88%), and excellent (90-100%) [5].

**Sample size:** the sample size was determined using Fischer´s formula for a cross-sectional study. Using the prevalence of presbyacusis of 2.5% from the study of Lasisi *et al*. [4], and 95% confidence interval and 5% degree of freedom this led to 37 as the minimum sample size, and after adding 10% for attrition sample size was 41.

**Sample strategy:** the participants were selected using the convenience sampling technique. Consecutive patients with presbyacusis who are 45 years and above and whom consented to participate in the study were screened by taking a clinical history and physical examination and subsequently had pure tone and speech audiometry. The external auditory canal was examined for wax, foreign body, discharge and state of the tympanic membrane. Inclusion criteria were adult-onset, bilateral, symmetrical (within 15dBHL), and sensorineural hearing loss, after excluding other causes of hearing loss. Participants with ear discharge, tympanic membrane retraction or perforation were excluded from the study, others excluded were patients with conductive or mixed hearing loss, and external ear deformity that can impair evaluation.

**Data analysis:** information obtained from the clinical and audiometric evaluation was recorded on a structured questionnaire and after verification entered into a computer. Data obtained was analysed using the statistical product and service solution (SPSS) version 20. Frequency tables and percentages were generated for categorical variables (gender, ethnicity, degree of hearing loss, type of presbyacusis) while mean and standard deviation were generated for continuous variables (age, hearing threshold, SRT and SDS). One sample binomial test was used to calculate the level of significance of gender, analysis of variance (ANOVA) was used to calculate statistically significant variance in the SDS in types of presbyacusis, and a student t-test was used to evaluate the significance of the different pairs of Schuknecht presbyacusis. The level of statistical significance was set at a p-value < 0.05.

**Ethical consideration:** the approval of the ethics and research committee of the hospital and Kaduna State Ministry of Health was obtained to conduct this study (as part of a broader research “Serum aldosterone in subjects with presbyacusis in Kaduna”) The consent of the participants in the study was obtained. The details of the procedure and the purpose of the study were explained to all the participants in the best language understood.

## Results

**Participants:** during the study period a total of 52 patients consented to be part of the study however 11 of these patients were excluded following clinical and audiometric evaluation due to the presence of either mixed hearing loss or asymmetric hearing loss that did not fit the diagnostic criteria for presbyacusis. The recruitment was completed after getting 41 participants which was 10% above the required minimum sample size calculated. The 41 participants selected, completed the study.

**Demographic characteristics:** the study involved 41 participants. The age range was from 45 to 85 years mean of 64.7±10.8 years. Twenty-eight (68.3%) were females while 13 (31.7%) were males, male-to-female ratio was 1:2 the difference was found to be statistically significant (p=0.029) using one sample binomial test. The study involved participants from 19 different tribes, majority were Hausa 12 (29.3%), Yoruba 6 (14.6%), and Edo 3 (7.3%).

**Pure tone audiometry:** the hearing threshold (calculated using pure tone average of 0.5, 1, 2 and 4 KHz frequencies) of participants´ ears ranged from 26.3 to 107.5 dBHL, mean of 55.7±18.2 dBHL. The hearing level increased steadily from the low to higher frequency (Table 1). The degree of hearing loss ranged from mild to profound hearing loss based on better hearing ears. The majority of participants 19 (46.3%) had moderate hearing loss, mild and severe hearing loss was found in 11 (26.8%) and 8 (19.5%) respectively while profound hearing loss was the least frequent of hearing loss was seen in 3 (7.3%) participants. For the configuration of the audiogram of 82 ears, moderate slope which signifies the neural presbyacusis was the commonest seen in 22 (28.8%) ears and the flat (metabolic or strial type) in 21 (25.6%) ears; gentle and steep slope constituted 19 (23.1%) and 10 (12.2%) of ears respectively signifying cochlear conductive and sensory presbyacusis respectively, therefore 51 (64.2%) of the ears had sloping configuration. Other configuration which included zig-zag 2 (2.4%) ears, ascending 3 (3.7%), ascending and descending 4 (4.9%) ears and descending- ascending configuration 3 (3.7%) ears constituted minority configurations (Table 2).

**Table 1:** mean decibel hearing level at different frequencies (air conduction) for subjects

Frequency (Hz)	Right ear	Left ear
	Mean± SD (dBHL)	Mean± SD (dBHL)
250	45.6±19.1	44.8±19.6
500	47.3±18.9	49.8±19.6
1000	54.6±6.4	53.3±22.4
2000	58.6±18.9	57.3±20.3
4000	62.8±21.2	60.5±22.0
8000	78.8±20.3	77.3±20.5

**Table 2:** configuration of audiogram and type of presbyacusis in ears of subjects

Configuration	Type of presbyacusis	Frequency (n)	Percentage (%)
Flat	Metabolic	21	25.6
Gentle slope	Cochlear conductive	19	23.1
Moderate slope	Neural	22	28.8
Steep slope	Sensory	10	12.2
Others	Others	10	12.2
**Total**		82	100.0

**Speech audiometry:** speech reception threshold of subjects ranged from 30-85 dBHL with a mean of 61.8±14.9 dBHL. The speech discrimination score (SDS), ranged from 0-98% with a mean of 69.3±20.0%. Only 8 ears (9.8%) of participants had excellent SDS, majority of ears of patients 30 (36.6%) had fair SDS. Good, poor, and very poor SDS were seen in 15 (18.3%), 7 (8.5%) and 22 (26.8%) of ears respectively. The SDS was highest in the sensory presbyacusis (83.8±7.7%) and lowest among the “others” presbyacusis (those that did not fit into the classical Schucknecht´s presbyacusis) 52.3±29.0% (Table 3).

**Table 3:** speech discrimination score versus configuration and types of presbyacusis

Configuration of audiogram	Type of presbyacusis	Speech discrimination score
		Mean±SD(%)
Flat	Metabolic	77.5±9.7
Gentle slope	Cochlear conductive	65.8±12.2
Moderate slope	Neural	69.4±23.7
Steep slope	Sensory	83.8±7.7
Others	Others	52.3±29.0
**SD**: standard deviation		

**Comparison of speech discrimination score using ANOVA:** analysis of variance of different types of presbyacusis revealed a statistically significant variance (F-value 112.45, a p-value of 0.000).

**Comparison of speech discrimination score in different pairs of Schucknecht´s presbyacusis using the 't' test:** SDS of sensory presbyacusis was found to be statistically significantly higher than the cochlear conductive, and neural presbyacusis (p-value 0.026 and 0.001 respectively) while the metabolic was higher than neural presbyacusis (p-value 0.005) (Table 4).

**Table 4:** comparison of speech discrimination score using student t-test in different pairs of Schucknecht’s presbyacusis

Comparison between types of Presbyacusis	‘t’ value	p-value
Metabolic versus sensory	-1.575	0.130
Metabolic versus cochlear conductive	1.239	0.224
Metabolic versus neural	3.014	0.005
Sensory versus cochlear conductive	2.365	0.026
Sensory versus neural	3.832	0.001
Cochlear conductive versus neural	0.584	0.563

t: student t-test. The level of statistical significance was set at a p-value < 0.05. Values colored green are statistically significant, while values colored black are not statistically significant.

## Discussion

The age range of 45-85 years was similar to the study by Sogebi *et al*. [10] which was 45-90 years and the study by Ogunleye *et al*. [11] (46-90 years), in this study, the youngest patient was 45 years similar to what Sogebi *et al*. [10] and Ogunleye *et al*. [11] found. The mean age of 64.7 years in this study was lower than that of Ogunleye *et al*. [11] which was 69.3 years, while Simonica *et al*. [12] in Brazil reported an age range of 40-86 years; with a lower mean of 50.5 years in comparison with this study, different studies have shown different age distribution of presbyacusis, this may be due to the difference in socio-demographics of their study population. In this study, elderly subjects 58.6% constituted the majority of participants similar to the study done by Sogebi *et al*. [10] of which elderly patients were the most prevalent constituting 53.6% of the patients, this was expected because presbyacusis is an age-related progressive disease [4,8].

In this study, the male-to-female ratio was 1: 2, this finding was similar to the study of Beraldi *et al*. [13] in Brazil who found female preponderance, but in contrast to the finding of Sogebi *et al*. [10] in Southwest Nigeria and Simonica *et al*. [12] in Brazil who found male preponderance. The study by Lasisi *et al*. [4] in Ibadan found more females but the difference was not statistically significant, Ogunleye *et al*. [11] also in Ibadan found more males, which was also not statistically significant. Beraldi *et al*. [13] in Brazil tried to explain why they found more females by associating it with the higher population of females in that age group in their City (Sao Paulo). Different studies have demonstrated different gender distributions; this may also be due to differences in socio-demographics of the study location.

Using better hearing to classify the degree of hearing loss, majority of the participants had moderate hearing loss (46.3%) and profound hearing loss was the least prevalent constituting 7.3% of participants. Sogebi *et al*. [10] and Fei *et al*. [14] also found moderate hearing loss as most prevalent at 26.1% and 29.5% respectively, although they had a lower percentage as compared to this study. In contrast to this study, Magalhães *et al*. [15] and McMahon *et al*. [16] found mild hearing loss as the most prevalent, Magalhães *et al*. [15] found 41% to have a mild degree of hearing loss while McMahon *et al*. [16] in a community longitudinal study found a much higher prevalence of mild hearing loss of 68.2%; considering the study of McMahon *et al*. [16] to be a community-based study they were likely going to discover the mild hearing loss, that may not present in the hospital because of the lower degree of handicap compared to moderate and severe degrees of hearing loss which would be seen in a hospital-based study.

In this study, higher frequencies were more affected than the lower frequencies but all frequencies were affected, this finding was similar to studies done by Sogebi *et al*. [10] where they found both speech and higher frequencies being affected, Beraldi *et al*. [13] in Brazil, Oh *et al*. [17] in Korea and Anjos *et al*. [18] in Brazil also found affectation of all frequencies with higher frequencies having poorer thresholds. The higher frequencies are more affected because they are represented at the basal end of the cochlear. Sensory, neural, and cochlear conductive types of presbyacusis affect the basal turn of the cochlear more, while the metabolic affect the apical and middle turn and sometimes the basal turn therefore generally presbyacusis affects the higher frequencies more, due to relative preservation of the apical part of the cochlea [3,12,19].

The moderate slope (neural presbyacusis) configuration was the commonest type of configuration seen in 22 (28.8%) ears followed by flat configuration (strial presbyacusis) in 21 (25.6%) ears. The neural type is considered to be the commonest type by Cheung *et al*. [3], in the study done by Sogebi *et al*. [10] and Ogunleye *et al*. [11] they found strial as the commonest configuration. Sarafraz *et al*. [20] found the sensory (61.1%) to be the commonest type. Sogebi *et al*. [10] reported 5 patients with symmetric hearing loss that didn´t fit into Shuknecht´s classical types, just like the finding in this study; the presence of these other configurations may be evidence of the indeterminate and mixed types of presbyacusis [3,12,16] Neural Presbyacusis results due to decrease in population of auditory neurons in cochlear and auditory nerve while the metabolic type is due to degeneration of stria vascularis. Different variety of presbyacusis have different prevalence across studies, and this may be due to the multi-factorial etiology of presbyacusis including both genetic and environmental factors [3,21-24].

The speech reception threshold (SRT) in this study, ranged from 30-85 dBHL with a mean of 61.8 dBHL, in a study done by Arlinger [25] Speech reception threshold ranged from a 22-93 dBHL mean of 45 dBHL in subjects with presbyacusis, Anjos *et al*. [18] in Brazil found mean SRT of 41.29±16.57 dBHL with a range of 16.57-110 dBHL; the higher mean in this study may be due to the higher degree of hearing loss and the multi-lingual nature of participants in this study.

In this study, the speech discrimination score (SDS) ranged from 0-98%, mean of 69.3±20.0%. Arlinger [25] found a range of 20-98% mean of 83%, while a study done by Anjos *et al*. [18] found a range of 0-100% and a mean of 75.99±22.66%, Beraldi *et al*. [13] found a range of 12-100% with a mean of 75.73% and 75.50% for the right and left ear respectively Beraldi *et al*. [13] associated the low mean in their study with higher degrees of hearing loss as well as the sloping configuration. The range of the speech discrimination score in this study was similar to the study of Beraldi *et al*. [13], Anjos *et al*. [18], and Arlinger [25], but this study had a lower mean than their studies; the lower mean in this study may be due to higher degree of hearing loss (majority had moderate hearing loss) and also the multi-lingual nature of subjects in this study. On comparing the speech discrimination scores in different presbyacusis the sensory presbyacusis had a better speech discrimination score than the neural presbyacusis as found in other studies [16,26]. It should be noted that in this study speech audiometry was done using an English language W-22-word list for all participants, therefore results may be poorer than their actual speech discrimination score considering the diversity of tribes.

## Conclusion

This study has found that neural presbyacusis was the commonest type of presbyacusis and moderate hearing loss was the commonest degree of hearing loss in presbyacusics, speech discrimination was affected in the majority of the subjects and sensory presbyacusis had the best speech discrimination score.

### What is known about this topic

- Presbyacusis has different pure tone audiogram configuration;
- Presbyacusis affects speech discrimination.

### What this study adds

- This study has added to the existing body of knowledge by discovering the different pure tone audiogram patterns of participants with presbyacusis in Kaduna, Northwest Nigeria, before this study only assumptions and extrapolations of this finding were done in this region, however, this study has shown that neural presbyacusis is the commonest and moderate hearing loss the most prevalent degree of hearing loss in a tertiary hospital setting;
- This study has confirmed the knowledge of the involvement of speech discrimination in presbyacusis and has shown the extent to which speech discrimination affects presbyacusis in Kaduna, North-West Nigeria;
- This study has also highlighted the need to develop a local language word list in testing speech discrimination, to get more accurate results.
